# Complications of subcutaneous penile modifications: a discussion of emergency department presentations and management

**DOI:** 10.1186/s12245-019-0241-z

**Published:** 2019-08-27

**Authors:** Cali L. Kirkham, Stormy M. Monks, Scott B. Crawford

**Affiliations:** 1 Texas Tech University Health Sciences Center El Paso Paul L. Foster School of Medicine, El Paso, TX USA; 2 Department of Emergency Medicine, Texas Tech University Health Sciences Center El Paso, 210 N. Rick Francis, El Paso, TX 79905 USA

**Keywords:** Subcutaneous penile modification, Pearl, Domino, Penis, Foreign body

## Abstract

**Background:**

Subcutaneous penile modifications (SPMs) are more prevalent in Southeast Asian culture and have been growing in popularity in Western culture. SPMs are often made of domino tiles, or other available pieces of plastic, shaved into a desired shape and placed in unsterile conditions. Previous literature indicates a high risk of infection and the need for surgical removal.

**Case presentations:**

Seven patients presented to the emergency department in the Southwest border region with complications from SPMs. All the patients complained of pain, four presented with signs of infection, and four SPMs required removal in the emergency department. Removal consisted of a dorsal penile nerve block and making an incision over the SPM to remove the foreign body. Three of the patients had their SPMs done during a previous incarceration under unsterile conditions.

**Conclusions:**

SPMs appear to be growing in popularity among Western culture, and emergency department health care providers should be aware of trends in body modifications as well as potential complications. The conditions in which SPMs are often placed pose a high risk for infection. In some cases, placement and/or removal of SPMs pose a risk of damage to the corpora, arteries, and nerves of the penis. In the absence of overt bleeding, or suggestion of neurologic injury, dorsally placed superficial foreign bodies of the penis may be amenable to emergency department removal.

## Background

Subcutaneous penile modification (SPM), also called “pearling” or “genital beading,” in Western culture is uncommon but may be growing in popularity along with tattoos, piercings, and other body modifications [1, 2]. Historically, the practice of penile modification is first mentioned in the Kamasutra as a method to enhance sexual pleasure [3, 4]. Recordings from Chinese explorers in Siam in the fourteenth and fifteenth centuries also documented jewels being inserted under the skin of the penis as a sign of wealth and for esthetic purposes [5].

Other authors have suggested that SPMs are common in Southeast Asian males [1]. The Japanese organized crime syndicate, Yakuza, is well-known for pearling—the process of inserting a pearl under the penile skin for each year in prison [2]. Aside from the Yakuza example, other commonly cited reasons for SPM are increased sexual pleasure for the individual and/or their partners and individual or sexual expression [6–9]. However, other studies have found that female partners of men with SPMs reported experiencing pain with intercourse [3, 6, 10] and difficulty using a condom [6].

The application of SPM generally requires small incisions on the shaft of the penis, where an object is then inserted subcutaneously [1, 8]. Previous case reports depict sharpened dominoes, glass beads, and pearls being inserted under the skin of the penis [8, 11]. This procedure is often not performed under sterile conditions, frequently presents with infection, and can require surgical removal [1, 10, 12]. Sharing of cutting implements such as razor blades has also been reported [6, 13]. In addition to men from Southeast Asia, many case reports of those with SPMs suggest that these men were also IV drug users, prisoners, or had gang affiliations [1]. One study found that men with SPMs were more likely to get paid for sex, have piercings, and, while in prison, get tattoos and take non-prescription drugs [13]. Another study found that men with penile modifications were more likely to contract a sexually transmitted disease [1]. Additional long-term complications of these procedures are unknown and understudied.

**Fig. 1:**
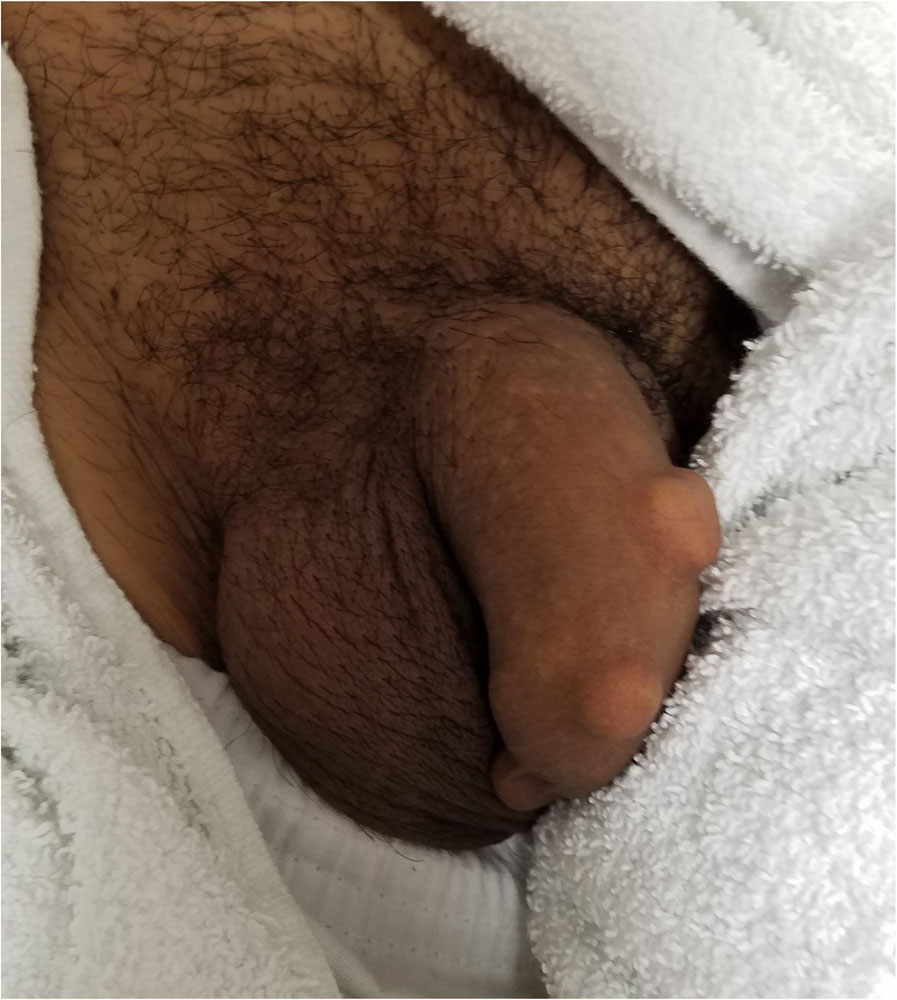
This image shows an example of a patient with multiple subcutaneous penile foreign bodies. In this case, three separate objects are visible and had been inserted by the patient. This patient presentation did not have any evidence of infection nor require intervention

**Fig. 2:**
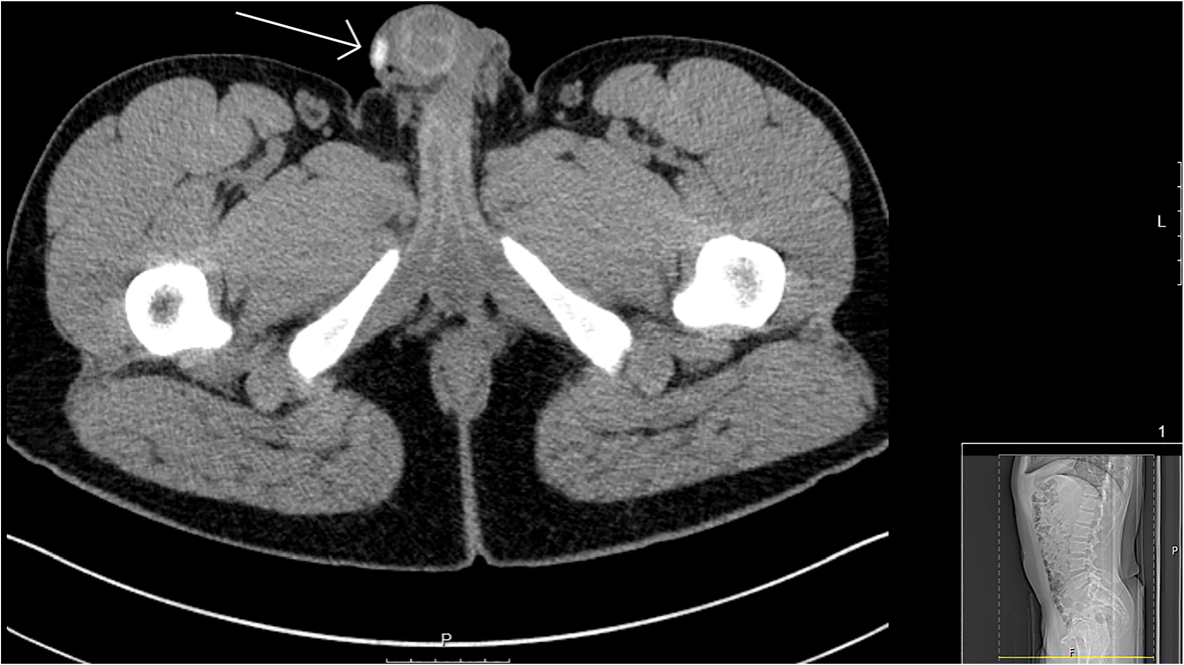
A computed tomography (CT) scan with radiopaque subcutaneous penile foreign body (white arrow) is present on this cut

A county hospital located along the U.S.-Mexico border with an annual census of 60,000 patients had seven patients present with complications of SPMs during a 5-year time period beginning in 2012. The majority of these SPMs were termed “dominoes” by the patients and several were specifically described as a tile from dominoes game and manually filed down on a concrete floor into a planned shape or design. Figure 1 and Fig. 2 depict the gross appearance and radiographic imaging of SPMs. Given the dearth of literature regarding SPMs in Western culture, this unique presentation should be documented for other emergency departments. This case series is meant to inform about growing trends, potential presentations, and serve as a guide for medical management in the emergency department setting.

## Case presentations

### Case 1

Patient 1 was a 28-year-old Hispanic male who presented to the emergency department with complaints of an infection of his penis. He reported making a dorsal slit and inserting a shaped domino 1 month prior. He stated that the pain was throbbing, did not radiate, and was growing in intensity for the week prior to arrival.

On physical exam, the patient had a punctate wound in the foreskin with pus and a foul smell coming from the wound. There was no evidence of cellulitis. The patient received a dorsal penile nerve block using 6 ml of 1% lidocaine, a small incision was made directly overlying the SPM, and a 5 mm × 1 cm bone-shaped domino was removed from under the foreskin using hemostats. He was discharged with a 10-day regimen of ibuprofen, cephalexin, and TMP-SMX DS.

### Case 2

Patient 2 was a 35-year-old Caucasian male who presented with complaint of discomfort due to a domino under the penile foreskin. He stated that the domino had been placed “surgically” 4 months prior to arrival, and it was now protruding from the skin. The patient denied any pain or signs of infection but requested removal.

Physical exam was unremarkable except for a 1 × 2.5-cm partially exposed foreign body at the original insertion site. He received a penile nerve block with 5 ml of 1% lidocaine and the foreign body was removed with minimal resistance. There was no purulent discharge. The patient was discharged with mupirocin 2% topical ointment and a 7-day regimen of cephalexin.

### Case 3

Patient 3 was a 39-year-old Hispanic male presenting with complaints of an infection to his penis. One year prior to arrival, the patient had a domino placed under the foreskin of his penis while incarcerated. On the day prior to ED arrival, he reported swelling, moderate pain, and reported that he had expressed pus adjacent to the foreign body.

His physical exam was unremarkable other than a heart-shaped foreign body under the penile skin of the right midshaft. There was no erythema, tenderness, weeping, or sign of recent infection. After examination, he was discharged with ibuprofen. Absent of infection or skin penetration, the decision was made to not remove the object.

### Case 4

Patient 4 was a 39-year-old Hispanic male presenting for pain management following surgical removal of SPMs. One day prior to arrival, he had surgery for an umbilical hernia repair and removal of three subcutaneous penile foreign bodies. According to the pathology report, surgeons removed three irregular faceted pieces of a yellow, hard, synthetic material ranging in size from 0.8 to 1.1 cm. Two of the pieces had tissue adhesions. Circumstances surrounding the placement of the SPMs are unknown.

The patient received acetaminophen with codeine and topical bacitracin as post-operative medications. After evaluation and pain control, no additional prescriptions were indicated.

### Case 5

Patient 5 was a 51-year-old Hispanic male who presented with complaints of a SPM shifting, causing pain and redness. He reported having an SPM placed while in prison 15 months prior to arrival. The SPM was fashioned from the handle of a toothbrush.

Physical exam was unremarkable except for a firm subcutaneous nodule at the distal dorsal penis at the ten o’clock position. Mild erythema and fluctuance was noted at the site of the SPM. A penile nerve block was performed by injecting a total of 5 ml of 1% lidocaine at the ten o’clock and two o’clock positions at the base of the penis and overlying the site of the foreign body. An irregular oval-shaped object of synthetic material measuring 1.5 by 0.7 by 0.3 cm was removed. He was discharged with a 10-day regimen of cephalexin.

### Case 6

Patient 6 was a 30-year-old Hispanic male with complaints of copious amounts of bleeding from his penis. He reported cutting his penis with a razor blade and attempting to place a domino while incarcerated.

On physical examination, the penis was noted to have swelling and tenderness to the dorsal shaft and a visible 2.5-cm superficial laceration. A 1-cm hard plastic foreign body in the shape of a swastika was removed and four 4–0 sutures placed. This patient was admitted after being given 1 g of ceftriaxone and placement of a Foley catheter. This management was performed in consultation with urology for concern of artery or nerve damage.

### Case 7

A 24-year-old male presented for evaluation of a chronic penile foreign body with an intermittent draining infection. The patient reported placing a piece of plastic from the handle of a toilet brush under the skin on the dorsum of his penis while incarcerated over 1 year prior. He described intermittent infections treated with oral antibiotics but had developed a persistent draining tract. Trace purulent drainage was noted without evidence of cellulitis. An ultrasound examination was performed to ensure the dorsal positioning was separate from the penile cavernosa. Due to the persistent soft tissue infection, the patient underwent bedside removal of the SPM.

**Fig. 3:**
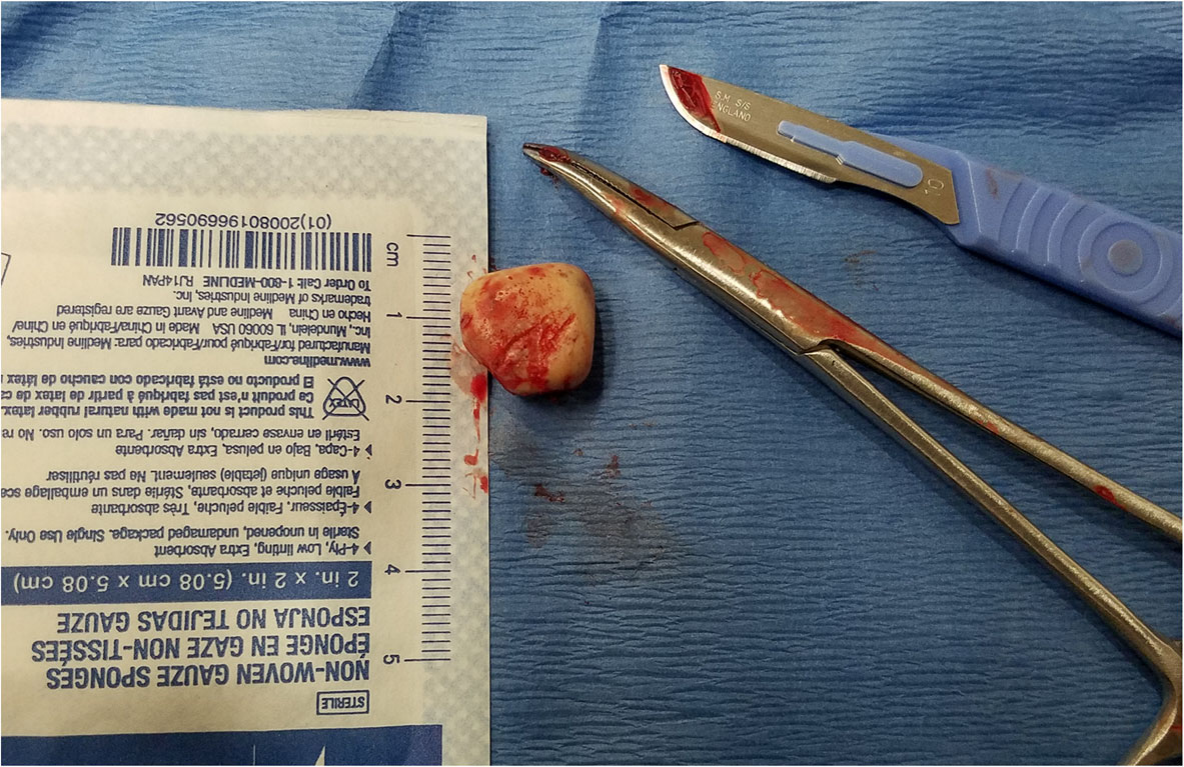
A photograph of an SPM that was removed due to persistent draining infection. The SPM was described by the patient as “Superman’s Crest”

This patient’s penis was cleansed with chlorhexidine, a penile nerve block was performed, and a 2-cm longitudinal incision was made overlying the SPM. Forceps were used for extraction of a 1-cm yellow tan plastic object (Fig. 3). The cavity was irrigated, loosely closed with 5–0 Vicryl sutures, and the patient observed to ensure bleeding was controlled prior to discharge. Cephalexin was provided with follow-up in 1 week for suture removal.

## Discussion

SPMs appear to be growing in popularity among Western cultures, particularly among prison populations. Emergency department health care providers should be aware of trends in body modifications as well as potential complications. Until SPMs are regularly performed in locations with standard safety and sanitation protocols, complications related to rejection and infection will continue to be seen in emergency departments.

While SPMs are generally more common in Southeast Asian males [3], a county emergency department located along the U.S.-Mexico border had a cluster of seven male patients with complaints related to SPMs. Four of the presentations had known prison connections with the placement of the SPM, consistent with previous findings [2, 9, 11, 13]. Conditions of the placement of the SPMs were reported to happen in non-sterile environments. Various methods were used for attempted cleansing and insertion. One patient reported cleaning a prison-issued razorblade with bleach and detergent before using it to insert a domino tile, another reported using a sharpened toilet brush handle as a “shiv” to poke a hole into the dorsum of his penis to allow SPM placement. More detailed information regarding the instruments used, whether or not the instruments had been shared, and the technique for SPM placement could give a more complete picture in terms of clinical risk and may help guide additional treatment or education to reduce morbidity.

Discomfort, infection, or infection risk was the documented reasons for removal of SPM in these patients, which is also consistent with previous findings [12]. Given that over half of the cases have a known incarceration history, information of other high-risk behaviors for infectious etiologies such as intravenous drug use and high-risk sexual behaviors can also be useful for determining clinical risk. Patients were treated with antibiotics targeted at local skin flora (TMP-SMX and cephalexin) if any signs of infection were present or if the clinician performed instrumentation of the area.

In addition to high-risk of infections, placement and removal of SPMs pose a risk of damage to the corpora, arteries, and nerves of the penis if care is not taken to avoid underlying anatomical structures. Emergency physicians should carefully evaluate for signs of damage from placement and consult urology if present. In particular, ventrally placed SPMs may pose a risk of urethral damage. Although SPM placement in this location was not encountered in this case-series, it has been reported elsewhere [14]. Additionally, injury to the tunica albuginea may lead to long-term complications such as impotence or abnormal curvature, similar to penile fracture, that may require surgical intervention [15]. To decrease the risk of injury to adjacent structures during removal, the authors suggest that only superficial and dorsally placed SPMs should be approached in the emergency department. Similar bedside removal has been previously described [8, 11]. An incision made directly overlying the SPM will minimize the risk of deep structure injury as the SPM can serve as a protective cutting barrier. When an infection is present, urgent removal of an SPM is indicated to prevent the progression of infection and further complications or tissue damage [16].

## Conclusion

This article will help clinicians have a better understanding of SPMs and the potential complications that may be associated with their placement, both acutely and chronically. SPMs may be encountered during routine radiographic imaging or physical examination and may not require any specific intervention. In the absence of overt bleeding, or suggestion of neurologic injury, dorsally placed superficial foreign bodies of the penis with evidence of infection may be amenable to emergency department removal. Questionable or high-risk cases should be referred to urology.

## Data Availability

Data sharing is not applicable to this article as no data sets were generated or analyzed during the current study. All research data access is limited to medical records contained within the county hospital EMR system where the patients presented.

## Abbreviations

SPM: Subcutaneous penile modification
